# DigiMir Test: Establishing a Novel Pipeline for MiR-371a Quantification Using Droplet Digital PCR in Liquid Biopsies From Testicular Germ Cell Tumor Patients

**DOI:** 10.3389/fonc.2022.876732

**Published:** 2022-06-10

**Authors:** José Pedro Sequeira, João Lobo, Vera Constâncio, Tiago Brito-Rocha, Carina Carvalho-Maia, Isaac Braga, Joaquina Maurício, Rui Henrique, Carmen Jerónimo

**Affiliations:** ^1^ Cancer Biology and Epigenetics Group, Research Center of IPO Porto (CI-IPOP)/RISE@CI-IPOP (Health Research Network), Portuguese Oncology Institute of Porto (IPO Porto)/Porto Comprehensive Cancer Centre (Porto.CCC), Porto, Portugal; ^2^ Master in Oncology, School of Medicine and Biomedical Sciences, University of Porto (ICBAS-UP), Porto, Portugal; ^3^ Department of Pathology, Portuguese Oncology Institute of Porto (IPOP), Porto, Portugal; ^4^ Department of Pathology and Molecular Immunology, School of Medicine and Biomedical Sciences, University of Porto (ICBAS-UP), Porto, Portugal; ^5^ Doctoral Programme in Biomedical Sciences, School of Medicine and Biomedical Sciences, University of Porto (ICBAS-UP), Porto, Portugal; ^6^ Department of Urology and Urology Clinics, Portuguese Oncology Institute of Porto (IPOP), Porto, Portugal; ^7^ Department of Medical Oncology and Urology Clinics, Portuguese Oncology Institute of Porto (IPOP), Porto, Portugal

**Keywords:** testicular germ cell tumors, ddPCR, hsa-miR-371a-3p, RT-qPCR, liquid biopsies, diagnosis

## Abstract

Testicular germ cell tumors (TGCTs) are the most common cancers in young-adult male patients aged between 15 and 39 years. Hsa-miR-371a-3p is currently the most reliable biomarker for diagnosis and monitoring of these patients non-invasively in liquid biopsies, and it is destined to be introduced in the clinic due to improved performance compared to the classical serum tumor markers available. Current studies have focused on real-time quantitative PCR (RT-qPCR) protocols for its determination; still, some challenges remain, since these protocols often require preamplification steps (costly and time-consuming), and report relative levels normalized to a housekeeping microRNA, not always performed the same way. Droplet digital PCR (ddPCR) shows the promise to overcome these challenges, skipping normalization and preamplifications, but has hardly been explored in the field of TGCTs. In this work, we provide a report of a ddPCR-based pipeline for the quantification of hsa-miR-371a-3p (the DigiMir pipeline) and compare it with two RT-qPCR protocols. A total of 107 plasma samples were investigated in the validation setting. The DigiMir pipeline detected TGCTs in a manner representative of tumor burden, with a sensitivity and specificity of 94% and 100%, respectively, outperforming the combined sensitivity of all three classical serum tumor markers (61.5%). Therefore, in this proof-of-concept investigation, we have shown that the DigiMir pipeline constitutes a new promising methodology to accurately report hsa-miR-371a-3p in the clinical setting.

## Introduction

MicroRNAs are small non-coding RNAs that are becoming more and more popular as biomarkers of disease, including cancer. They are interesting for clinical use in part due to their stability in bodily fluids, making them attractive non-invasive liquid biopsy biomarker candidates, but also due to ease of detection with relatively low-cost methodologies widely available, such as PCR-based ones (1). These microRNAs are also dynamic and versatile, reflecting the status of disease and often being useful for diagnostic, prognostic, and follow-up purposes. However, still, few of these promising microRNAs actually make it to full integration in the clinic, in part due to technological challenges related to detection and reporting (2).

Testicular germ cell tumors (TGCTs) are among the most common solid neoplasms arising in young-adult Caucasian men (3). For these tumors, a set of microRNAs that regulate embryonic development have proved their value as accurate liquid biopsy biomarkers of the disease (4–6). Among them, the hsa-miR-371a-3p has shown the best clinical results, outperforming the classical serum tumor markers nowadays available in the routine [alpha fetoprotein (AFP), human chorionic gonadotropin (HCG), and lactate dehydrogenase (LDH)], which show important limitations (7–11). Since the study of Voorhoeve et al. in 2006 (12), an overwhelming amount of evidence has been built in the last decade, demonstrating the accuracy of hsa-miR-371a-3p, determined by real-time quantitative PCR (RT-qPCR), for the diagnosis and follow-up of TGCT patients [except for pure teratoma (13)], with sensitivities and specificities of mostly >90% (14–17). This microRNA has a short half-life, correlates with tumor burden, can be reliably detected in several bodily fluids, and is able to predict recurrences and viable tumor in post-chemotherapy masses (18–27). The confirmation of these findings in large, prospective, multicentric studies increased even more the interest in this biomarker (28, 29), leading to organization of clinical trials (NCT03067181 and NCT04435756) and proposals for introduction of a quantitative test in the clinic (30, 31).

However, there is still room to attempt to improve such a test. An increased technical sensitivity is desirable for stage I patients with low tumor burden, sometimes missed by the hsa-miR-371a-3p test, not only for diagnosis but especially for early discrimination of patients at risk of relapsing, allowing the timely adjustment of the therapeutic strategy (surveillance versus adjuvant chemotherapy), sparing young patients from unnecessary cytotoxic treatments (32, 33). Moreover, most protocols for the determination of hsa-miR-371a-3p levels rely on preamplification steps, which may be argued to produce some variability related to increased cycling (besides being costly, time-consuming, and facilitating events of contamination and unspecific amplifications). Also, the commonly used protocols rely on normalization to housekeeping microRNAs (variable from study to study), giving a relative quantification (as opposed to the absolute number of copies of the marker), and the method of normalizing and reporting RT-qPCR data differs among studies, raising the need for a consensus procedure and pipeline (31, 34).

In recent years, droplet digital PCR (ddPCR) has emerged as a new methodology for accurately quantifying liquid biopsy biomarkers and for the detection of minimal residual disease (35, 36). DdPCR provides absolute quantification, obviating the need for normalization to housekeeping microRNAs and facilitating setting of cutoffs of positivity, and is less influenced by inhibitory substances, due to the partitioning process. However, its application for microRNA quantification is still largely unexplored [with few studies available (37–44)], and only one very recent study attempted to apply such protocol to hsa-miR-371a-3p determination in TGCTs (45).

In this work, we describe a pipeline for the quantification and reporting of hsa-miR-371a-3p in plasma samples of TGCT patients using ddPCR (*DigiMir test*) and provide a direct comparison with two other available RT-qPCR methods for the quantification of this biomarker. We perform several technical optimizations of the ddPCR pipeline, which obviated the preamplification step, and finally validate our findings in a larger cohort of plasma samples.

## Methods

### Samples

A total of five plasma samples were included in the proof-of-concept phase of the study, where the three pipelines were compared and the optimization of technique was performed: two stage I seminomas, one stage II embryonal carcinoma, one stage III embryonal carcinoma, and a young-adult male healthy blood donor.

After optimization of the ddPCR pipeline, a validation set of 107 plasma samples was investigated, comprising 56 samples from TGCT patients (31 drawn right before orchiectomy and 25 in the context of routine follow-up), three samples from patients with non-TGCT testicular masses (two Leydig cell tumors and one Mullerian type tumor of the testis), 47 male healthy blood donors, and additionally one patient with an AFP-secreting hepatocarcinoma (representing the context of non-TGCT-related AFP elevation). Of the 31 TGCT patients included in the study, all 31 had a pre-orchiectomy sample, six had one follow-up sample, eight had two follow-up samples, and one patient had an additional intermediate sample, totaling three follow-up samples. The median time from orchiectomy to the first follow-up samples was 6 months (interquartile range [IQR] = 8 months); the median time from orchiectomy to the second follow-up samples was 15 months (IQR = 7 months). Also, all blood samples coincided with routine determinations of classical serum markers AFP, HCG, and LDH, which were available and compared to hsa-miR-371a-3p measurements.

All patients were diagnosed and treated at IPO Porto by the same multidisciplinary team. Patients with suspicion of other malignancies were excluded. After the collection of peripheral blood into EDTA-containing tubes, plasma was separated centrifuging at 2,500 rpm for 30 min at 4°C, and subsequently stored at −80°C in the institutional tumor bank. All blood samples were processed within 4 h maximum from collection. All clinical and histopathological data of the patients were reviewed by a TGCT-dedicated pathologist and according to the most recent WHO 2016 classification and AJCC 8th edition staging manual (46).

Complete description of the optimization and validation cohorts is provided on **Table 1**.

**Table 1:** Clinicopathological description of the optimization and validation cohorts.

Optimization cohort (n = 5 samples)	
Cases	Description
Sample #1	37 years, Seminoma, Stage I
Sample #2	28 years, Seminoma, Stage I
Sample #3	37 years, Embryonal carcinoma, Stage II
Sample #4	34 years, Embryonal carcinoma, stage III
Sample #5	44 years, healthy blood donor
**Validation cohort summary (*n*= 107 samples)**	
TGCT samples	56
Pre-orchiectomy	31
First follow-up timing	15
Second follow-up timing	9
Third follow-up timing	1
Non-TGCT testicular mass samples	3
Male healthy blood donors	47
Non-testicular tumor with elevated AFP (hepatocarcinoma)	1
**TGCT patients—clinicopathological features**	
Age (years [median, interquartile range])	33 (8.5)
Size of tumor mass (cm [mean, interquartile range])	5.7 (3.95)
Histology (*n*, %)	
Seminoma	19/31 (61.3)
Embryonal carcinoma	2/31 (6.5)
Mixed tumor	9/31 (29.0)
Postpubertal-type yolk sac tumor	1/31 (3.2)
Stage (*n*, %)	
I	19/31 (61.3)
II	6/31 (19.4)
III	6/31 (19.4)
AFP positive (*n*, %)	9/31 (29.0)
HCG positive (*n*, %)	12/31 (38.7)
LDH positive (*n*, %)	14/31 (45.2)
Either AFP or HCG or LDH positive (*n*, %)	20/31 (64.5)

AFP, alpha fetoprotein; HCG, human chorionic gonadotropin; LDH, lactate dehydrogenase.

### RNA Extraction

Total RNA was extracted from 100 µl of plasma and eluted in 50 µl of elution buffer, using the MagMAX miRvana Total RNA Isolation kit (Thermo Fisher, A27828), according to the manufacturer’s protocol. As a technical control, the non-human synthetic spike-in ath-miR-159a (0.2 µl per sample of a stock solution at 0.2 nM) was added to the lysis buffer (with 5’-Phosphate modification for TaqMan Advanced microRNA protocol, see below).

### TaqMan Advanced (Global) MicroRNA Pipeline

Two microliters of isolated RNA was reverse transcribed and preamplified (14 cycles) using the Taqman Advanced hsa-miRNA cDNA synthesis (Thermo Fisher, A28007) in a Veriti™ 96-Well Thermal Cycler (Applied Biosystems™), following the manufacturer’s protocol. Then, 2.5 µl of the diluted preamplification product was plated on 96-well plates and run on a QuantStudio 12K Flex platform using the following conditions: 5 µl of TaqMan Fast Advanced Master Mix (2×), 0.5 µl of TaqMan Advanced hsa-miRNA Assay (20×), and 2 µl of RNase-free water; assays: ath-miR-159a—478411_mir, FAM; hsa-miR-371a-3p—478070_mir, FAM; hsa-miR-30b-5p—478007_mir, VIC; run conditions: 95°C for 20 s followed by 40 cycles at 95°C for 1 s and 60°C for 20 s.

### TaqMan (Target-Specific) MicroRNA Pipeline

Five microliters of isolated RNA was reverse transcribed for the following pool of microRNAs (ath-miR-159a, hsa-miR-371a-3p, and hsa-miR-30b-5p) using the TaqMan microRNA Reverse Transcription kit (Thermo Fisher, 4366596) in the same Veriti™ thermocycler, according to the protocol reported in (18). Next, 5 µl of cDNA was preamplified (12 cycles) using the TaqMan Preamp Master Mix (Thermo Fisher, 4391128), and then eluted in 75 µl of bidistilled water. One microliter of the diluted preamplification product was plated on the same QuantStudio 12K Flex platform using the following conditions: 5 µl of 2× TaqMan Universal Master Mix no UNG, 0.5 µl of TaqMan hsa-miRNA Assay (20×), and 3.5 µl of RNase-free water; assays: ath-miR-159a—000338, FAM; hsa-miR-371a-3p—002124, FAM; hsa-miR-30b-5p—000602, VIC; run conditions: 50°C for 2 min, 95°C for 10 min, followed by 40 cycles at 95°C for 15 s and 60°C for 1 min.

### Droplet Digital PCR: DigiMir Pipeline

Five microliters of isolated RNA was reverse transcribed for the following pool of microRNAs (ath-miR-159a and hsa-miR-371a-3p) using the TaqMan microRNA Reverse Transcription kit (Thermo Fisher, 4366596) in the same Veriti™ thermocycler, according to the protocol reported in (18). DdPCR reactions were prepared as follows: 2 µl (ath-miR-159a) or 4 µl (hsa-miR-371a-3p) of cDNA, 11 µl of ddPCR Supermix for probes (Bio-Rad, California, USA, #1863010), 1 µl of TaqMan hsa-miRNA Assay (20×), and 8 µl (ath-miR-159a) or 6 µl (hsa-miR-371a-3p) of bidistilled water; assays: ath-miR-159a—000338, FAM and hsa-miR-371a-3p—002124, FAM. Droplets were generated on the automated droplet generator QX200 AutoDG (Bio-Rad, California, USA) and read on the QX200 Droplet Reader (Bio-Rad, California, USA). The PCR run was set as follows: 95°C for 10 min, 50 cycles of 94°C for 30 s and 56°C for 1 min—ramp rate 2°C/s—and 98°C for 10 min.

The limit of blank (LOB) and limit of detection (LOD) of the assays were calculated as indicated in (47). The limit of quantification (LOQ) was assessed by performing a 2-fold dilution series of a TGCT sample for both miRNAs.

### Quality Control Steps and Statistics

Plasma samples were visually inspected, and no samples had obvious signs of hemolysis. Appropriate engineering and manual controls were used to prevent contaminations, including master mix made using a clean and UV-irradiated hood prior to adding any template, clean gloves, PCR reagents, and consumables, and reactions were performed in separate dedicated labs. RNA was extracted from the seminoma-like cell line TCam-2 and used as a positive control for hsa-miR-371a-3p in all pipelines. No template control (NTC) and no enzyme control (NEC) were included in all cDNA synthesis and PCR stages, as negative controls. For ddPCR pipeline optimization, further negative controls (“no cDNA control”, “no Supermix control”, and “no assay control”) were included, as recommended (44). All RT-qPCR reactions were performed in triplicate, and those in ddPCR were performed in duplicate. Results were plotted in GraphPad Prism 9. The thresholds of positivity of the hsa-miR-371a-3p and ath-miR-159a assays were defined based on LOD calculation, as determined below. Correlations were computed using the non-parametric Spearman correlation coefficient. Diagnostic performance of the DigiMir pipeline was assessed by calculating sensitivity, specificity, negative predictive value (NPV), and positive predictive value (PPV) for diagnosis of TGCT.

**Figure 1:**
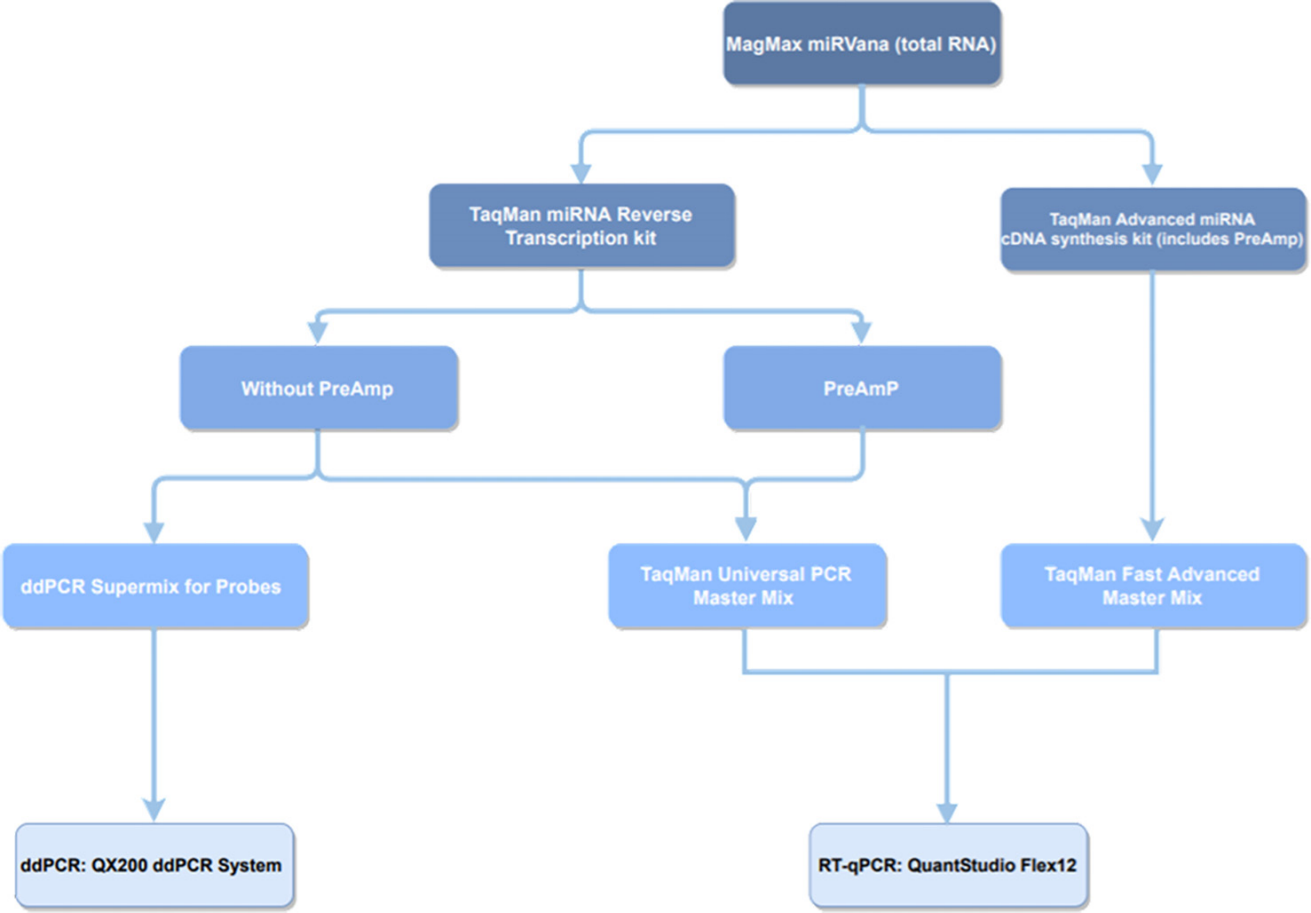
Diagram of the study protocol, including RT-qPCR and ddPCR pipelines. ddPCR, droplet digital PCR; preamp, preamplification; RT-qPCR, real-time quantitative PCR.

A diagram of the study protocol is provided in **Figure 1**.

## Results

### Optimization Phase and Comparison of Pipelines

#### Input and Temperature Settings

We first optimized the input of the PCR reactions, specifically in the TaqMan (target-specific) microRNA protocol. We found no remarkable differences in Ct values obtained for synthetic ath-miR-159a when using either 1 µl or 2 µl of cDNA as input (Cts 13.940–14.285 and 13.873–14.130).

**Figure 2:**
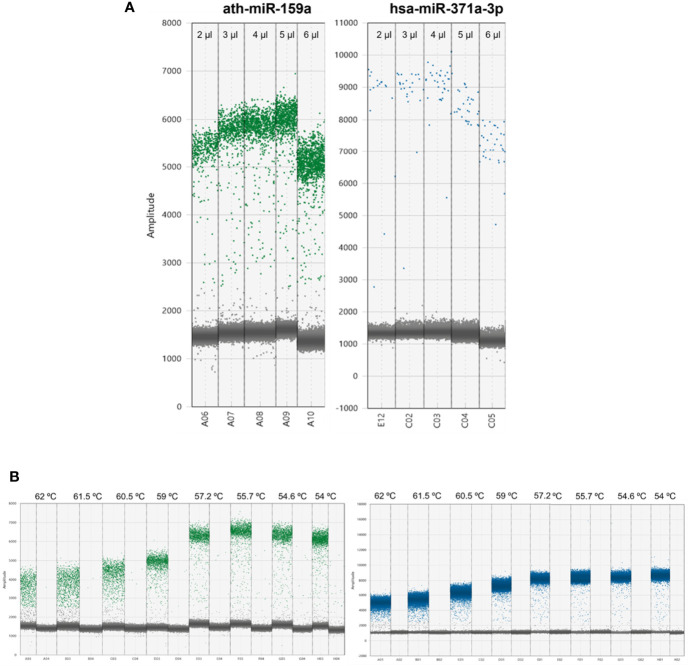
**(A)** Optimization of DigiMir pipeline. Ath-miR-159a and hsa-miR-371a-3p input optimization. **(B)** Temperature gradient for further optimization of ath-miR-159a (green) and hsa-miR-371a-3p (blue) assays on the DigiMir pipeline. Notice that the best separation is achieved approximately 56°C for both assays.

For the DigiMir pipeline, and to achieve accurate separation of positive and negative droplets for the ath-miR-159a, the optimal input was determined to be 2 µl, which rendered an optimal number of positive droplets (**Figure 2A**). The optimal input for hsa-miR-371a-3p was determined to be 4 µl, which rendered more positive droplets when compared to 2 µl and 3 µl and, approximately, the same positive droplets when compared to 5 µl and 6µl.

These optimized input conditions were used for the following tasks.

A temperature gradient (56–62°C) was then run to further optimize the droplet separation of the protocol. We verified that a temperature of approximately 56°C (55.7°C) provided the best separation (less rain) and highest amplitude for both ath-miR-159a and hsa-miR-371a-3p, and this was set for the validation study (**Figure 2B**). The optimal amplitude thresholds of 2,500 were applied to achieve maximal separation for both ath-miR-159a and hsa-miR-371a-3p.

#### Controls, Sensitivity, and Specificity

Related to non-specific amplifications with both RT-qPCR-based protocols, we found the TaqMan Advanced (global) microRNA pipeline to result in “undetermined” Ct for the healthy blood donor (and in the TCam-2 sample, which was not spiked-in with the synthetic oligo), but to produce sporadic late amplifications for ath-miR-159a in the NTC inserted on cDNA synthesis (Ct 33.3) and for hsa-miR-30b-5p in the NEC (Ct 37.5).

For the TaqMan (target-specific) microRNA protocol, in the runs performed for optimization, we could detect non-specific late amplification for the hsa-miR-371a-3p in the male healthy blood donor (Ct 30.5) and in the NTC inserted on cDNA synthesis (Ct 38). Also, non-specific amplifications were detected for both hsa-miR-30b-5p and ath-miR-159a in the NTC inserted in cDNA synthesis (Ct 32 and Ct 29.5, respectively) and in the NEC (Ct 34 for both). The ath-miR-159a also rendered a non-specific late amplification in the non-spiked-in TCam-2 sample (Ct 38).

For the DigiMir protocol and using the thresholds of positivity defined below for both hsa-miR-371a-3p and ath-miR-159a, the healthy male blood donor sample was negative for the hsa-miR-371a-3p. The remaining negative controls (NTCs, NECs, and, additionally, “no cDNA control”, “no Supermix control”, and “no assay control”) were consistently negative for hsa-miR-371a-3p and for ath-miR-159a considering the calculated LOB.

**Figure 3:**
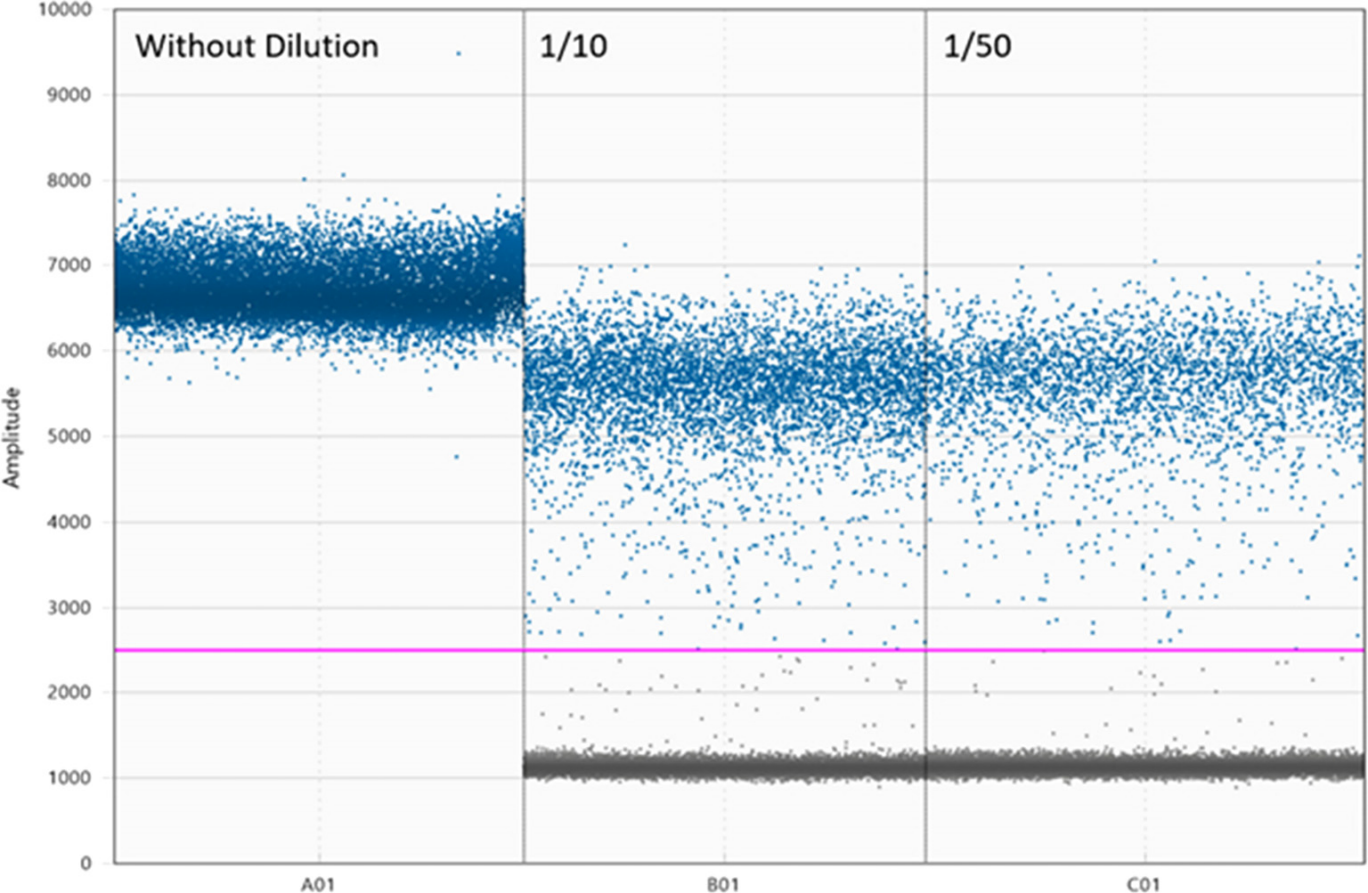
Optimization of DigiMir pipeline. Best separation of positive and negative droplets in the hsa-miR-371a-3p TCam-2 positive control with 1:50 dilution. Notice that there is no separation of droplets in the undiluted sample.

As for the TCam-2 positive control, the TaqMan Advanced (global) microRNA pipeline detected hsa-miR-371a-3p at Ct 14.5, while an earlier Ct of 8 was produced with the TaqMan (target-specific) microRNA protocol. For the DigiMir pipeline, and to achieve accurate separation of positive and negative droplets, TCam-2 serial dilutions were performed, with the best separation being set at 1:50 (**Figure 3**).

**Figure 4:**
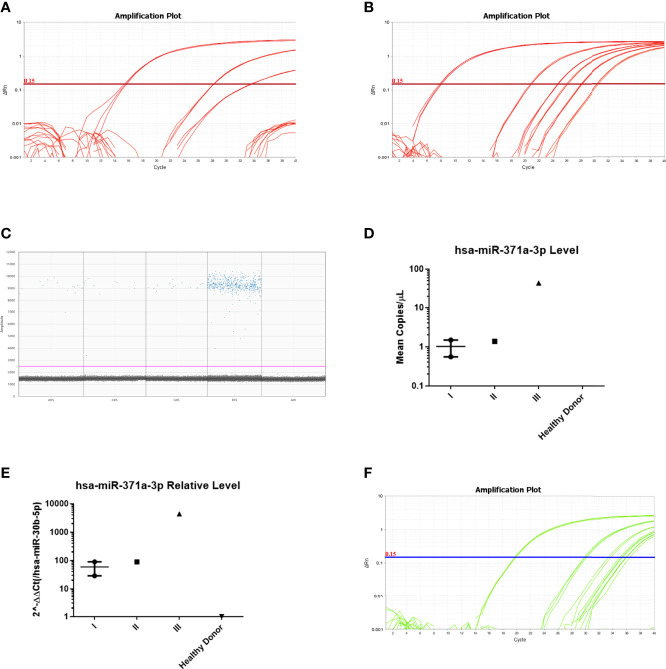
Detection of patient samples by PCR methodologies. **(A)** Detection of samples of the optimization phase using the TaqMan Advanced (global) microRNA pipeline. Notice the amplification of positive control TCam-2, followed by the stage III patient and one of the stage I patients. The remaining two patients (and the healthy blood donor) are not amplified. **(B)** Detection of the same samples using the TaqMan (target-specific) microRNA pipeline. Notice the overall earlier amplification of all samples. The positive control TCam-2 is detected, followed by all patient samples (stage III, stage I, stage II, and stage I from left to right). However, the healthy blood donor also shows amplification (curve most to the right). **(C)** Detection of the same samples using the ddPCR pipeline. Notice the overall amplification of all samples. All patient samples (stage I, stage I, stage II, stage III, and healthy donor from left to right) amplified. **(D)** Absolute levels of hsa-miR-371a-3p using the ddPCR pipeline. **(E)** Relative levels of hsa-miR-371a-3p, normalized to hsa-miR-30b-5p levels, using the TaqMan (target-specific) microRNA pipeline. **(F)** Same samples and protocol used in B, but omitting the preamplification step. Notice that all samples amplify later, and only the TCam-2 and the stage III patient (curves most to the left, respectively) are detected with a Ct value lower than 34.

Related to the ability of detection of small burden stage I disease, we verified that with the TaqMan Advanced (global) microRNA pipeline, we could only detect hsa-miR-371a-3p in one of the two stage I seminoma samples (**Figure 4A**). There was a positive correlation with tumor burden, with higher relative levels being detected in stage III compared to stage II and stage I disease.

However, both stage I samples could be detected with TaqMan (target-specific) microRNA protocol and with ddPCR pipeline (**Figures 4B–D**). A positive correlation with tumor burden was also shown by both protocols, with higher levels in stage III compared to stages II and I (**Figure 4D** for the ddPCR pipeline and **Figure 4E** for RT-qPCR).

We also performed the TaqMan (target-specific) microRNA protocol omitting the preamplification step, with a 2-µl input in the PCR run. The stage III sample was detected (Ct 30.0), but both the stage I and stage II samples gave only late amplifications (Ct >34), evidencing the reduced sensitivity for detecting low burden disease without preamplification in the RT-qPCR method (**Figure 4F**).

#### Variability and Precision

**Figure 5:**
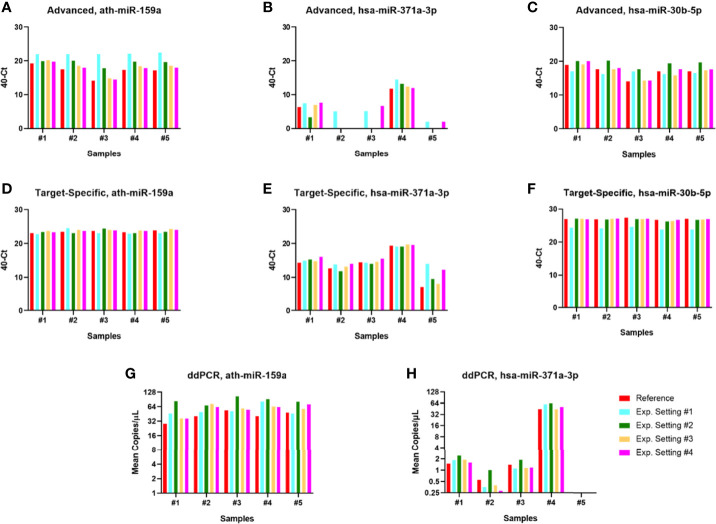
Comparison of pipelines. **(A–C)** TaqMan Advanced (global) microRNA pipeline. **(D–F)** TaqMan (target-specific) microRNA pipeline. **(G, H)** DigiMir pipeline. The *x*-axis represents the samples used for the optimization phase: #1 and #2—stage I seminomas; #3—stage II embryonal carcinoma; #4—stage III embryonal carcinoma; #5—age-matched healthy blood donor. The color code refers to experimental settings: setting #1—different extraction; setting #2—different cDNA synthesis; setting #3—different operator; setting #4—same operator; and reference for comparison.

To assess the variability of the three pipelines, we defined four experimental settings: “Setting #1”, with cases being extracted in two distinct timings; “Setting #2”, where the same extraction was always used but cases were submitted to two different cDNA syntheses; “Setting #3”, with samples deriving from the same extraction and cDNA synthesis, but with two different operators plating the PCR reaction; and “Setting #4”, same as Setting #3 but with the same operator plating the PCR reaction twice, on two distinct occasions (**Figure 5**).

The TaqMan Advanced (global) microRNA pipeline showed the highest variability between the various experimental settings, as observed in **Figures 5A–C**. This included the assessment of the non-human spike-in ath-miR-159a, which had poorer recovery (later Ct range) when compared to the TaqMan (target-specific) microRNA protocol and resulted in higher variability in spike-in detection among samples. The overall higher reproducibility of the TaqMan (target-specific) microRNA protocol is evidenced in **Figures 5D–F**.

For the DigiMir pipeline, ath-miR-159a was well recovered in all samples (**Figure 5G**) and hsa-miR-371a-3p was detected in all experimental instances, illustrating tumor burden (with higher detection in the stage III sample) and being negative (below the threshold of positivity) for the healthy blood donor (**Figure 5H**).

**Figure 6:**
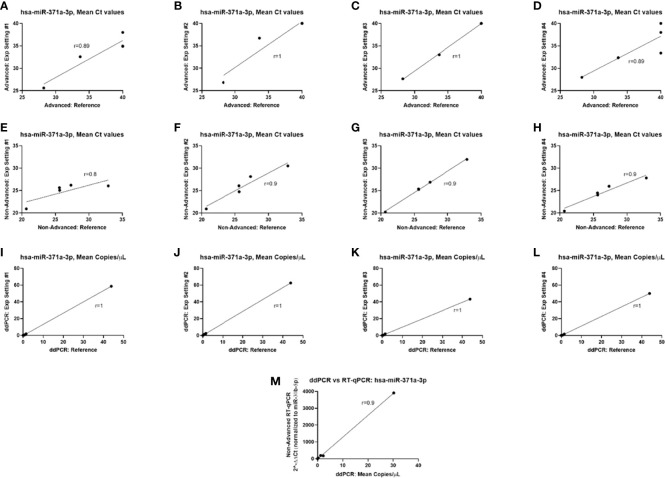
Correlation between hsa-miR-371a-3p quantifications. **(A–D**) TaqMan Advanced (global) microRNA pipeline. **(E–H)** TaqMan (target-specific) microRNA pipeline. **(I–L)** DigiMir pipeline. **(M)** Correlation between the DigiMir pipeline and the TaqMan (target-specific) microRNA pipeline quantifications (the latter normalized to hsa-miR-30b-5p and using the 2^−ΔΔCt^ method).

When comparing the results for hsa-miR-371a-3p quantification between the various experimental settings, a strong positive correlation was found for all instances (**Figures 6A–L**). Additionally, when comparing the quantification of hsa-miR-371a-3p using the TaqMan (target-specific) microRNA protocol with the one obtained with the DigiMir pipeline, a strong positive correlation between hsa-miR-371a-3p relative levels (normalized to hsa-miR-30b-5p) and absolute copies/µl was obtained (**Figure 6M**).

#### Further Optimization of ddPCR Protocol

To determine the LOB and LOD, 30 NTC samples inserted in the cDNA synthesis step were run for both hsa-miR-371a-3p and ath-miR-159a. The LOB of the hsa-miR-371a-3p was then determined to be 3 droplets, meaning an LOD of 5 droplets. The LOB of ath-miR-159a was determined to be 14 droplets, meaning an LOD of 21 droplets. The LODs of the assays were set as thresholds of positivity for the corresponding assays in this pipeline.

**Figure 7:**
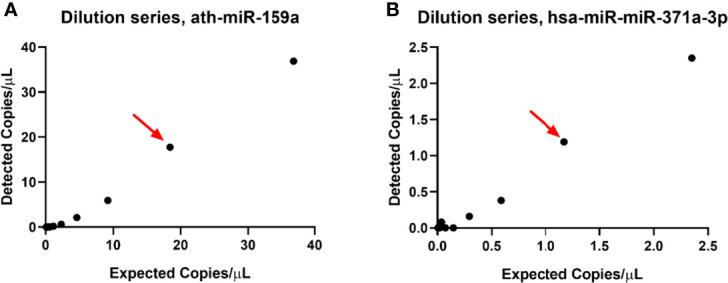
Limit of quantification of ath-miR-159a **(A)** and hsa-miR-371a-3p **(B)** assays. The red arrow points to the number of copies that the assay can still reliably quantify.

To calculate the actual LOQ (allowing for precise quantification of the absolute number of copies present in the sample), 2-fold dilution series of a positive case were performed as indicated above. The LOQ was determined to be 18.4 copies/µl for ath-miR-159a and 1.17 copies/µl for hsa-miR-371a-3p (**Figure 7**).

A summary of the comparison among the three protocols is provided in **Table 2**.

**Table 2:** Summary of the three different pipelines for comparing hsa-miR-371a-3p.

Variables	MagMax miRvana extraction, spike in ath-miR-159a, 100 µl plasma		
	Thermo Fisher, Advanced (global)	Thermo Fisher, non-Advanced (targeted)	DigiMir, Bio-Rad (targeted)
Preamplification step	Mandatory	Advised (better results)	Accurate detection without preamp
Reaction volumes	No difference in Cts between using 1 µl or 2 µlNeed for triplicates		More sensitivity with 4 µl Duplicates suffice
Spike-in recovery	Higher variability, poorer recovery 0.2 µl of 1 nM	Lower variability, better recovery 0.2 µl of 1 nM	Good recovery 0.2 µl of 0.2 nM
Normalization (miR-30b-5p)	Needed ΔΔCt		Not needed Precise absolute number of copies/µl
Healthy blood donor	Undetermined	Sporadic amplification	Negative
Detection of stage I SE	+/- (1/2)	+ (2/2)	
Tumor burden representation	Yes (stage III >>> stage I)		
Technical Variability	Overall poorer	Adequate	
NTC inserted on cDNA synthesis	Hsa-miR-371a-3p: Undetermined ath-miR-159a: sporadic amplification	Hsa-miR-371a-3p: sporadic late amplification ath-miR-159a: late amplification	Hsa-miR-371a-3p: Negative ath-miR-159a: Negative
NEC inserted on cDNA synthesis	Hsa-miR-371a-3p: Undetermined ath-miR-159a: Undetermined	Hsa-miR-371a-3p: Undetermined ath-miR-159a: sporadic amplification	Hsa-miR-371a-3p: Negative
NTC inserted on PCR plate	Hsa-miR-371a-3p: Undetermined Hsa-miR-30b-5p: Undetermined ath-miR-159a: Undetermined		Hsa-miR-371a-3p: Negative ath-miR-159a: Negative
Positive control	Adequate Hsa-miR-371a-3p: Cts +-14.5	Adequate Hsa-miR-371a-3p: Cts +-8	Adequate Hsa-miR-371a-3p: optimal dilution for separation 7is 1:50

### Clinical Validation of the DigiMir Pipeline

There were 19 seminoma and 12 non-seminoma samples. The median age of the TGCT patient cohort was 33 years, and the one of the male healthy blood donors was 46 years. Most patients were stage I TGCTs (61.3%). AFP, HCG, and LDH were elevated at the time of diagnosis (pre-orchiectomy) in 29%, 38.7%, and 45.2% of patients. Elevation of any of the classical serum tumor markers was found in 64.5% of TGCT patients (**Table 1**).

Using the DigiMir pipeline, hsa-miR-371a-3p outperformed the classical serum tumor markers, achieving a sensitivity of 93.6%, a specificity of 100%, an NPV of 96%, a PPV of 100%, and an accuracy of 97.5% for identifying TGCT patients in the pre-orchiectomy setting. No healthy blood donors showed detectable hsa-miR-371a-3p above the defined cutoff, and only two TGCT patients were negative for hsa-miR-371a-3p, corresponding to two seminomas (also negative for all three classical serum tumor markers). Additionally, the three non-TGCT testicular masses were negative for hsa-miR-371a-3p, as was the hepatocarcinoma patient with elevated AFP. Hsa-miR-371a-3p levels were significantly positively correlated with levels of HCG, LDH, and tumor size (*r* = 0.57, *p* < 0.001; *r* = 0.544, *p* = 0.002; and *r* = 0.475, *p* = 0.007, respectively).

Regarding the follow-up samples of TGCT patients, they were all negative in patients with no signs of disease after the orchiectomy, with normalization of classical serum tumor markers and negative imaging scans. Of note, the single patient with stage III and S1 disease after orchiectomy (with persistent HCG elevation) also showed elevation of hsa-miR-371a-3p 10 days after the orchiectomy. The patient was treated with 4xBEP but showed only partial response and is now with disease progression (elevation of AFP, HCG, and *de novo* supraclavicular lymphadenopaties). One patient with a seminoma but with slight AFP elevation pre- and post-orchiectomy (interpreted clinically as a constitutional elevation of this marker) was decided to be put on surveillance; remarkably, the hsa-miR-371a-3p was negative in the post-orchiectomy follow-up samples of this patient.

**Figure 8:**
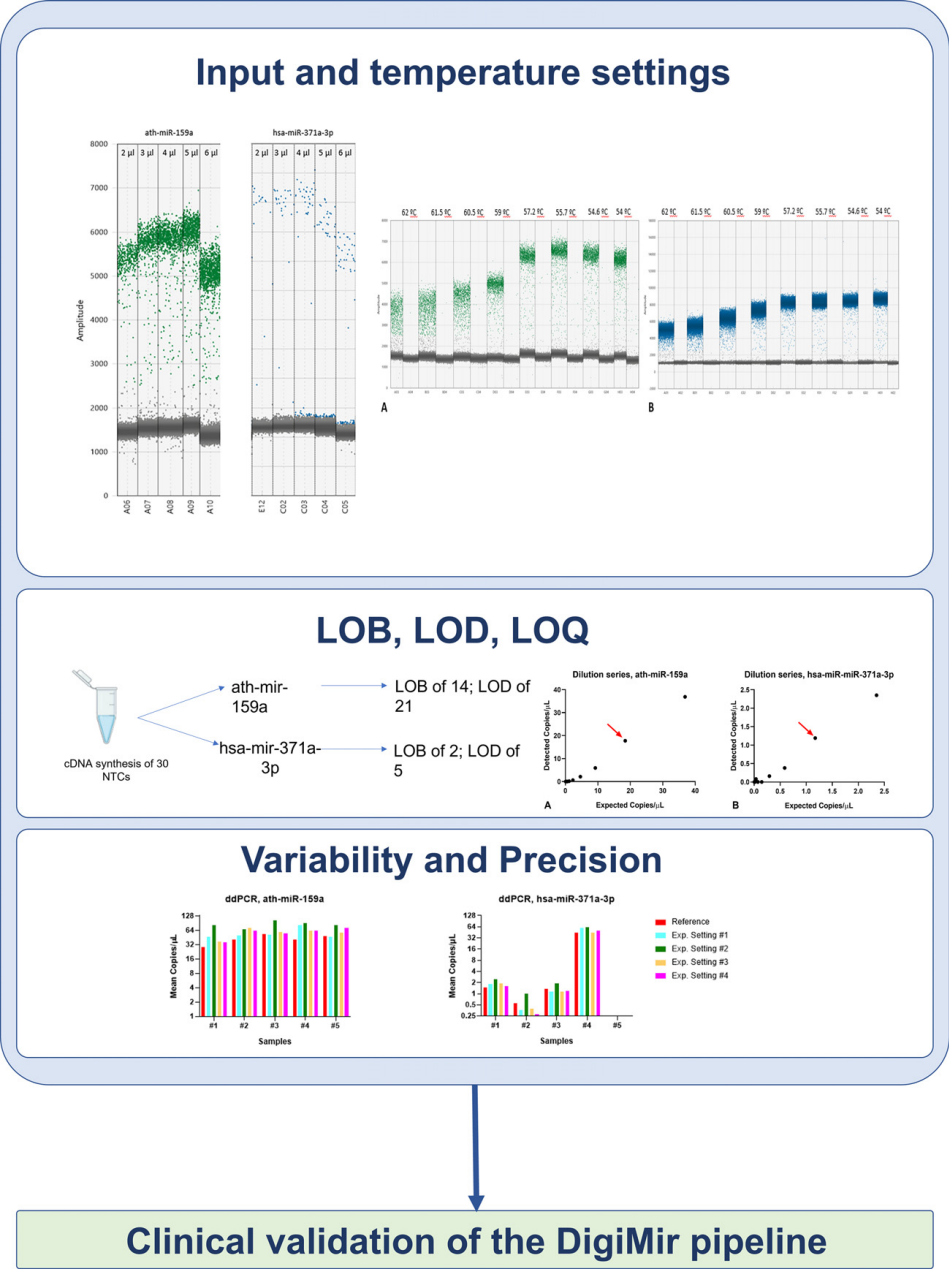
DigiMir pipeline optimization steps.

A summary of the several steps undertaken for the optimization of the DigiMir pipeline is illustrated in **Figure 8**.

## Discussion

In this work, we describe a pipeline for quantifying the most remarkable liquid biopsy biomarker in the field of TGCTs based on ddPCR, being approximately 3 times cheaper than RT-qPCR-based techniques. In fact, and despite an increased interest on ddPCR for accurately determining biomarkers in samples with low burden and low input (like in circulating markers such as microRNAs and cell-free DNA in plasma), few studies have decided to invest on microRNA detection using this technology (42) [the same for circulating tumor cells (48)], perhaps because the more conventional RT-qPCR-based techniques have been performing well in this task, at low cost and with more disseminated know-how. DdPCR has emerged as a very robust way to determine mutation status in circulation (49), and also as a way to study DNA methylation patterns in circulating cell-free DNA (50). Studies on microRNAs are very recent, and report different protocols for achieving distinct needs. Tavano et al. reported a protocol to determine hsa-miR-1290 for the diagnosis of pancreatic cancer in plasma using ddPCR (37), with this method being superior to routine CA19-9 determination, while Zhao and collaborators investigated ddPCR to determine a panel of microRNAs diagnostic of gastric cancer (40). Campomenosi and co-workers compared RT-qPCR with ddPCR for the assessment of several microRNAs in serum of lung cancer patients and demonstrated a strong positive correlation between the two techniques, with equal or smaller variation in the ddPCR approach (38). This high concordance between the two methods reported by the authors led us to seek a similar methodological approach for our study. Indeed, we have performed back-to-back comparison of three independent pipelines for quantifying hsa-miR-371a-3p. Two of the protocols were RT-qPCR-based (the TaqMan Advanced method, where a global microRNA synthesis with preamplification is performed, and the TaqMan non-Advanced method, with targeted microRNA synthesis for the desired microRNAs and preamplification). While both pipelines produced similar results, the TaqMan Advanced methodology [less reported in literature specifically in the field of TGCTs (23)] produced more variability in the recovery of non-human spike-in ath-miR-159a and resulted in a lower sensitivity of detection of low burden stage I cases. We hypothesize that this could be explained by global preamplification (instead of targeted reaction) interfering with sensitivity of detection (51). For these reasons, the targeted approach for cDNA synthesis was considered more reliable and was chosen to translate into ddPCR quantification. Nevertheless, both RT-qPCR protocols detected hsa-miR-371a-3p in a manner consistent with disease burden, with the stage III patient rendering higher levels of hsa-miR-371a-3p.

In this work, we attempted to omit the preamplification stage by stepping-up to the ddPCR methodology. Preamplification may theoretically introduce bias by increased cycling and is cumbersome and frequently the most expensive section of the experimental protocols, but is highly adopted in most recent works in the field [as summarized in (31)], but was not performed in older works (9), because of the increasing sensitivity of detection of low amounts of circulating microRNA, such as in stage I small tumors. However, it can also elicit sporadic detection of trace amounts of hsa-miR-371a-3p in teratoma-only cases or even in healthy subjects, which are undesirable and could create discomfort when reporting the results—also evidenced in this work. In this study, we have optimized the DigiMir pipeline (after fulfilling ddPCR technical requirements and precision assessment, such as assuring the greatest separation of positive and negative droplets, temperature gradient, LOB and LOD calculation, and dilution series, as required and reported (39, 41, 44) and were able to quantify hsa-miR-371a-3p absolute copy number in a manner consistent with tumor burden (higher in stage III), with all negative controls and the healthy blood donor being below the threshold of positivity, hence being considered negative.

We designed experimental settings to illustrate variability in quantification of microRNAs with the three distinct pipelines, again with the TaqMan Advanced RT-qPCR protocol demonstrating to be poorer, with higher variability in results upon the various repetitions of the run under different conditions. Shifting to ddPCR, recovery of spike-in was homogeneous among samples [within the range reported in (39)] and variability of detection of hsa-miR-371a-3p was acceptable, with a strong positive correlation found between the amounts determined at varied moments [as reported also in (39)]. Most importantly, like Campomenosi (38), we also found the quantification results by RT-qPCR (normalized to housekeeping hsa-miR-30b-5p) and those of ddPCR (with the advantage of obviating normalization) to be strongly positively correlated.

Then, in an early attempt to provide clinical validation, we have applied the defined DigiMir pipeline to a set of 107 plasma samples, including those from TGCT patients, non-TGCT testicular masses, healthy male blood donors, and one patient with AFP secretion due to a hepatocarcinoma. Remarkably, hsa-miR-371a-3p identified all but two TGCT patients (which were also negative for AFP, HCG, and LDH), and correctly discriminated all 47 healthy blood donor male individuals as negative. The resultant specificity of 100% and a sensitivity of 94% are similar or even superior to the values reported in previous RT-qPCR-based studies (52) [summarized in (34)], outperforming the combined sensitivity of the three classical serum tumor markers (61.5%). Indeed, there were 11 TGCTs negative for all three classical serum tumor markers available in routine, which were, however, detected by our assay, representing an important clinical benefit in diagnosis. Furthermore, as additional negative controls from a clinical standpoint, the three non-TGCT testicular masses were negative for hsa-miR-371a-3p, a point in favor of the specificity of our assay. Non-TGCT masses are not infrequent and constitute an important differential diagnosis of TGCTs, which can however only be confirmed after orchiectomy is performed (53). Importantly, for small benign tumors, partial orchiectomy may be an option, allowing to spare fertility and function of the testis (54). A large study already reported that such masses are negative for hsa-miR-371a-3p by RT-qPCR (55), and we show the same by ddPCR approach. Additionally, our assay has proved to be useful in clarifying elevations of AFP due to causes other than TGCTs, as has been recently reported for RT-qPCR (56). The patient with an important elevation of AFP due to a hepatocarcinoma was negative (with 0 copies) for hsa-miR-371a-3p. Also, of interest, a seminoma patient showed an elevation of AFP pre-operatively, which he maintained post-orchiectomy and during follow-up. The elevation was interpreted clinically as constitutional since no further disease could be identified, but caused discomfort in clinical decisions, especially for the decision to put the patient on surveillance only. In this setting, our hsa-miR-371a-3p assay would have been useful to the clinic, since it was positive at pre-orchiectomy (due to the presence of the seminoma), but completely negative (0 copies) after orchiectomy despite the persistence of AFP, confirming that the elevation was constitutional or derived from other causes.

Finally, we also tested an additional setting of relevance, which is the follow-up of these patients (57). Follow-up routinely involves repeat measurements of classical serum tumor markers (which show important limitations in detecting relapses) and continuing imaging [both costly, with limited sensitivity for very small metastatic deposits and exposing young patients to radiation if computed tomography is performed (30, 58, 59)]. In this sense, hsa-miR-371a-3p measurements are attractive for adequate monitoring and for guiding treatment decisions (34). In our study, all follow-up samples from patients showing disease resolution and no signs of disease recurrence (negative imaging and classical serum tumor markers) were negative for hsa-miR-371a-3p, while the follow-up sample of a patient with stage III S1 disease was positive.

The data we present are overall corroborated by the very recent study of Myklebust et al. (45), who also describe a ddPCR pipeline for determining hsa-miR-371a-3p. However, there are important differences in methodology between the two studies: (1) whereas they used serum, we tested plasma samples, which may have an impact on microRNA determination, namely, on the levels of the housekeeping microRNA miR-30b-5p (18); (2) the RNA extraction kits and methodologies differed; and (3) Myklebust and co-workers did not include a spike-in (technical control) in their experimental setting, whereas, in our pipeline, spike-in recovery was demonstrated in all experiments as quality control (ath-miR-159a). Nevertheless, the main results of both studies completely concur in that ddPCR is a promising new methodology for reporting has-miR-371a-3p, with high sensitivity (89% for the authors and 94% in our study) and specificity (100% in both works) and associating with tumor burden. Additionally, both investigations report a good correlation and technical performance in comparison with RT-qPCR, having the advantage of obviating the preamplification step and allowing to report absolute copy numbers (without the need of a standard curve) in a clinical setting. Specifically, our study brings the novelty of including a seminoma with constitutional elevation of AFP. In our view, this further supports the relevance of our findings and suggests that the DigiMir pipeline will be also useful in the follow-up setting. Validation in larger cohorts of TGCT patients on surveillance, as performed in other studies (33), is warranted and would contribute to clarify the clinical utility of our assay.

To conclude, we provide a proof-of-concept investigation describing a methodology to quantify the most relevant liquid biopsy biomarker of TGCTs in plasma samples, disclosing adjustments to the protocol and technologies that can make the quantification less variable and more precise, achieving an accuracy of 97.5% in a validation cohort of 107 samples. A limitation of the study is the small patient cohort, not allowing to focus on specific settings such as stage I tumors with recurrence, or <1-cm masses. However, this constituted a first clinical validation of our pipeline, which we technically describe in detail. Further investigations and validation of the protocol are warranted in different clinical contexts. In particular, it will be interesting to investigate if ddPCR may improve the follow-up of stage I patients on active surveillance, or aid in the assessment of marker-negative small testicular masses and viable germ cell malignancy in the post-chemotherapy metastatic context, to better personalize treatment and monitoring of TGCT patients (60).

## Data Availability Statement

The original contributions presented in the study are included in the article/supplementary material. Further inquiries can be directed to the corresponding authors.

## Ethics Statement

This study was approved by the Ethics Committee (CES-IPO-12-018) of Portuguese Oncology Institute of Porto, Portugal. The patients/participants provided their written informed consent to participate in this study.

## Author Contributions

JPS, JL, and VC performed molecular analyses and wrote the manuscript. JL collected the clinical data. TB-R assisted in specific tasks related to molecular analyses. JPS, JL, and VC analyzed the data. CC-M processed clinical samples. JM provided clinical information about the patients. RH and CJ supervised the work and revised the manuscript. All authors contributed to the article and approved the submitted version.

## Funding

The authors would like to acknowledge the support of the Programa Operacional Competitividade e Internacionalização (POCI), in the component FEDER, and by national funds (OE) through FCT/MCTES, in the scope of the project EpiMarkGermCell (PTDC/MEC-URO/29043/2017). The authors would also like to acknowledge the support of MSD (“Prémio de Investigação em Saúde”), Banco Carregosa/Secção Regional do Norte da Ordem dos Médicos (SRNOM), and Fundação Rui Osório de Castro/Millennium bcp. JL is the recipient of a fellowship from FCT—Fundação para a Ciência e Tecnologia—(SFRH/BD/132751/2017). VC received the support of a fellowship from “la Caixa” Foundation (ID 100010434). The fellowship code is LCF/BQ/DR20/11790013.

## Conflict of Interest

The authors declare that the research was conducted in the absence of any commercial or financial relationships that could be construed as a potential conflict of interest.

## Publisher’s Note

All claims expressed in this article are solely those of the authors and do not necessarily represent those of their affiliated organizations, or those of the publisher, the editors and the reviewers. Any product that may be evaluated in this article, or claim that may be made by its manufacturer, is not guaranteed or endorsed by the publisher.
